# Impact of Participation in Role-Playing Game (RPG) Sessions on the Perceived Level of Social Anxiety and Received Social Support

**DOI:** 10.3390/brainsci15111158

**Published:** 2025-10-28

**Authors:** Zdzisław Kroplewski, Roksana Łoś, Bartłomiej Józef Pawlicki

**Affiliations:** 1 Department of Social Sciences, University of Szczecin, 71-017 Szczecin, Poland; zdzislaw.kroplewski@usz.edu.pl; 2 Electrical Department, West Pomeranian University of Technology in Szczecin, 71-313 Szczecin, Poland; pawlicki.paw@wp.pl

**Keywords:** social anxiety, social support, role-playing games

## Abstract

**Background/Objectives:** Anxiety disorders are among the most common mental disorders and are often associated with significant discomfort and impaired functioning. One of the more frequent forms is social anxiety disorder, which is characterized by excessive fear of social evaluation and the avoidance of social situations. The aim of this study was to assess the potential of role-playing games (RPGs) as an alternative form of support for people with social anxiety disorder. **Methods**: Thirty participants aged 18–28 with a non-generalized form of social anxiety were qualified for the study and assigned to two conditions differing in session frequency (once a week vs. once every two weeks). Participants were assigned to groups based on the order of registration for the study. As the recruitment was open to the public and participants registered voluntarily, the assignment process was not strictly random and may have been influenced by self-selection factors. The intervention lasted 3 months and included elements of social exposure and social skills training within a structured RPG scenario. The study lasted from March to November 2024 at the Faculty of Social Sciences of the University of Szczecin. Standardized tools (LSAS, ISSB) were used to measure social anxiety and received social support before and after the intervention. Statistical analyses were performed using the JASP statistical program version 0.17.2. **Results**: The results indicate a statistically significant reduction in anxiety and avoidance in all groups, with a greater effect observed in the once-a-week group (Cohens’s d = 0.94). At the same time, an increase in perceived social support was noted, especially in the biweekly condition. The greatest changes were observed in the total support score, while specific components (emotional, informational, instrumental) showed differentiated dynamics depending on frequency. **Conclusions**: The findings suggest that RPG-based interventions may serve as a preliminarily effective and engaging form of support for individuals with social anxiety, contributing to symptom reduction and improved functioning in social contexts.

## 1. Introduction

Anxiety disorders are one of the main categories of mental disorders, the essence of which is inadequate emotional reactions resulting from difficulties in adapting to the environment or a specific situation. The result of these disorders is often significant psychological discomfort and cognitive impairment [1]. Social anxiety disorder is characterized by intense and persistent fear or anxiety, triggered by one or more social situations in which the individual may be observed by other people. This fear is most often associated with the fear of negative social evaluation, embarrassment, or humiliation. People suffering from this disorder often avoid social situations or endure them with high tension and anxiety, leading to a significant deterioration in their functioning in the social sphere [2]. An important diagnostic criterion is the disproportionality of the perceived fear in relation to the actual threat and its persistence for at least six months. Avoidance of social situations or intense fear of them causes clinically significant functioning disorders in social, educational or professional areas [3].

Previous studies indicate significant negative correlations between the severity of anxiety symptoms and the tendency to avoid social situations in the course of social anxiety disorder and the level of social support received [4]. These results suggest that social support may play an important buffering role in the symptoms of this disorder. The concept of social support is multidimensional and includes various forms of interpersonal interaction. The mechanisms of its impact on the functioning of an individual are complex and may include emotional, informational and instrumental aspects [5].

The concept of role-playing as a therapeutic form in the treatment of anxiety disorders was first described as early as the 1940s. Andrew Salter, in his work *Conditioned Reflex Therapy* [6], pointed to the potential of this method in the context of behavior modification. Since then, numerous studies have been published on the use of role-playing methods in therapy, for example, Social Skills Training (SST), in the treatment of anxiety disorders, many of which have been empirically verified in recent years [7].

In the literature on the subject, four basic components of social skills are distinguished [8]: non-verbal skills—including eye contact, facial expressions, gestures, body posture and interpersonal distance; verbal content—referring to the adequacy of the utterance, and the accuracy of the message in relation to the situation and the topic of conversation; paralinguistic features—such as the tone, pitch and intensity of the voice, and intonation, fluency and clarity of speech; interactive balance—including the ability to react to the interlocutor, and the length of the speech and reaction delay. Social Skills Training can therefore be defined as the systematic practice of the behaviors that make up the above components. SST is a flexible method that can be adapted to the individual needs and abilities of the participants, which translates into the effectiveness of this method [9]. The most common form is group classes, which are conducive to modeling behavior and learning through observation and interaction with other participants.

In recent years, there has been growing interest in using gamification as an innovative approach not only to therapy but also to mental health prevention, both in children and adults. New interventions are designed based on recognized therapeutic methods, such as the above-described Social Skills Training, the exposure method, cognitive–behavioral therapy, as well as gamification mechanisms, which promotes their accessibility, scalability, and participant engagement [10]. In this context, special importance is given to co-op role-playing games, known as role-playing games (RPGs) or TTRPGs (Tabletop Role-Playing Games). These are multiplayer games based on social interaction, collaborative decision-making, and active role-playing, conducted according to pre-established rules [11]. Games of this type allow you to modify their structure and adapt the elements of the game to the individual needs of the participants. Another important advantage is the ability to react in real time, use reward systems, score, and monitor progress, which is conducive to increasing involvement in the therapeutic process. Preliminary research results suggest that RPGs can support the development of social skills, increase emotional self-awareness, and support the internalization of new, more adaptive behavior patterns [10].

Although the therapeutic potential of RPGs is increasingly being described in the international literature, most research to date has been exploratory or descriptive. To date, most analyses of RPGs in mental health settings have been based on case studies or small qualitative studies, and only a few studies have systematically measured pre- and post-intervention outcomes [12]. Furthermore, data indicate that RPGs can be used as an active intervention aimed at reducing anxiety. In a pilot study [13] involving six participants, they found that RPG sessions have promising therapeutic potential for addressing anxiety symptoms. Research also indicates that role-playing games provide a safe, structured environment conducive to practicing social skills and developing socio-emotional competencies and can be easily adapted for interventions for anxiety disorders [14]. Current literature [15,16,17] indicates that the use of role-playing games is an effective intervention not only for social anxiety, but also for reducing depressive symptoms, social isolation, and working with individuals on the autism spectrum—both in clinical settings (as a complement to cognitive–behavioral therapy) and non-clinical settings. These games create a safe environment in which participants can confront situations they previously avoided and develop empathy and emotion regulation skills.

Difficult situations created by the “Game Master” can reflect participants’ everyday experiences [16]. Through role-playing and supportive group dynamics, players have the opportunity to reframe maladaptive behavior patterns into more constructive ones and transfer them to real-life functioning. Collaborative narrative creation fosters bonding, group solidarity, and a sense of security, which provides a foundation for emotional and social change [18]. The literature also highlights the similarity of RPGs to techniques used in Social Skills Training (SST), where role-playing is a key element [19]. RPGs can serve as a transitional stage in exposure therapy, enabling the simulation of anxiety scenarios in a safe environment [20]. Although some researchers report that participation in these games promotes the development of assertiveness, conflict resolution, emotion regulation, and interpersonal communication, despite a growing number of publications, few studies address the actual effectiveness of these interventions [21].

In the Polish context, research on the use of role-playing games as a form of psychological or social support is still very limited. Although RPGs are gaining popularity as a form of entertainment and social activity, their targeted use in a therapeutic or paratherapeutic context has not yet been empirically investigated. This study aims to fill this gap by assessing the potential of RPG sessions as an alternative form of support for individuals with social anxiety disorder. By collecting pre- and post-intervention data from 30 participants, this study represents one of the largest quantitative studies in this field, in the context of research on the use of TTRPGs in the treatment of social anxiety disorder in the Polish setting.

The aim of this study is to assess the potential of role-playing games as an alternative method to support the treatment of social anxiety disorder. It is assumed that the use of this form of intervention can contribute to the reduction of anxiety symptoms and avoidance of social situations, as well as act as social support by enabling safe and controlled participation in group interactions. This is a pre–post single-group design with different frequency conditions.

## 2. Materials and Methods

### 2.1. Subjects

This study was reviewed and obtained ethical approval from the Ethical Committee for Research Projects of the Institute of Psychology, University of Szczecin, number KB6/2024. A total of 30 people were qualified for the study, including 13 women and 17 men aged 18–28. The inclusion criteria for the study were student status and a LSAS score of 30–60, which indicated non-generalized social anxiety disorder. Exclusion criteria included participation in any form of anxiety disorder therapy during the study period. Participants were assigned to groups based on the order of registration for the study. As the recruitment was open to the public and participants registered voluntarily, the assignment process was not strictly random and may have been influenced by self-selection factors. Participants were assigned to groups of five, each containing at least two women, to exclude all-male or all-female groups. However, assignment to a specific group was determined primarily by the order in which the applications were received.

### 2.2. Demographic Data

Data collection included demographic information, such as participants’ age and gender, place of residence, current field of study, and familiarity with the activity of RPGs. These data are included in Table 1. The study involved 13 women and 17 men, students living in the city of Szczecin.

Study participants were aged 18–28 years, and the prevalence of individuals of each age is also presented in Table 1. These students were studying psychology (1), English studies (2), business administration (3), mathematics (4), law (5), automation and robotics (6), computer science (7), Norwegian studies (8), interior design (9), applied linguistics (10), management (11), and electrical engineering (12). All participants confirmed that they knew what RPGs were, but until the study began they had never actively participated in them.

### 2.3. Procedure

Before participating in the experiment, the subjects were given a consent form to voluntarily participate in the study and were informed about the purpose of the study, its course, and the possibility of withdrawing from the study at any time. In the first part of the study, participants were asked to complete two questionnaires examining the level of received social support (Inventory of Socially Supportive Behaviors—ISSB) and the level of experienced social anxiety (Liebowitz Social Anxiety Scale—LSAS), as well as a self-report survey. In the second part of the study, participants were divided into 6 teams, each consisting of 5 participants. Each team met for 3 h over a period of 3 months. Teams 1A, 1B and 1C met once a week (hereinafter referred to as Condition A1), while Teams 1D, 1E and 1F met once every two weeks to investigate the possible effect of training frequency (hereinafter referred to as Condition A2). During the meetings, participants experienced both social exposure and social skills training to the extent allowed by the chosen scenario. Participants played a fragment of the “Call of Cthulhu: Masks of Nyarlathotep” adventure scenario [22], modified for the research (fewer combat-initiating situations, more situations requiring cooperation and leveraging the strengths of the characters created by the participants). Each group began with the prologue of the scenario, and the extent to which they implemented it depended on the quality of their cooperation. In the third part of the study, participants were asked to re-complete the questionnaires from the first part of the study. The study lasted from March to November 2025 at the Faculty of Social Sciences of the University of Szczecin.

### 2.4. Statistical Analysis

In order to answer the research questions statistical analyses were performed using the JASP statistical program version 0.17.2. The study involved a detailed analysis of basic descriptive statistics, including means, standard deviations, and normality of distribution. Then, comparisons of measurement results were performed between groups, taking into account each of the analyzed variables separately. For each data set, the value of the *t*-test for independent samples as well as Cohen’s d coefficient were calculated, which allowed not only the assessment of the statistical significance of differences but also the estimation of their effect size. This made it possible to comprehensively capture both quantitative and qualitative aspects of differences between the study groups. Due to the relatively small sample size, additional power analyses (post hoc tests) were performed. The results suggest that the social anxiety measures were sufficiently powered, while the social support measures—especially in the less frequent group—would have required a larger number of participants. A report of the power analyses is available in the Supplementary Materials.

## 3. Results

Analysis of the ISSB scores before the intervention shows that higher mean values of social support were observed in the groups from condition A2 (group meetings once every two weeks). Table 2 presents descriptive statistics for the ISSB test results prior to participation in the experiment.

The analysis of the ISSB values after participation in the study indicates an increase in the received social support in both conditions. Greater improvement was observed in group A2 (group meetings once every two weeks). Table 3 presents descriptive statistics for the ISSB test scores after participation in the study.

Analysis of the level of social anxiety before the intervention shows that a higher level of anxiety was noted in group A1 (group meetings once a week). The results of the Shapiro–Wilk test indicate that the groups in condition A2 (group meetings once every two weeks) show deviations from normality (*p* < 0.05), which suggests that the results in this condition should be interpreted with caution. Table 4 presents descriptive statistics for the Liebowitz Social Anxiety Scale scores prior to participation in the study.

Analysis of the results after the intervention indicates a significant reduction in social anxiety in all groups. A greater reduction was noted in the groups from condition A2 (group meetings once every two weeks). Table 5 presents descriptive statistics for the results of the Liebowitz Social Anxiety Scale after participation in the study.

Table 6 presents the results of the dependent samples *t*-test for the measures of the four aspects of social support (emotional support, informational support, instrumental support, and evaluative support) and the overall score measure in the two conditions (before and after participation in the study) of the groups from condition A1, i.e., participating in group sessions once a week. All tests, except the measure of evaluative support, have *p* values < 0.05, suggesting significant differences between conditions. The evaluative support score (*p* = 0.095) does not reach statistical significance. The overall outcome measure (*p* = 0.016, d = −0.618) has the largest effect size, meaning that the difference between pre- and post-study scores is not only significant but also has a medium effect size.

**Figure 1:**
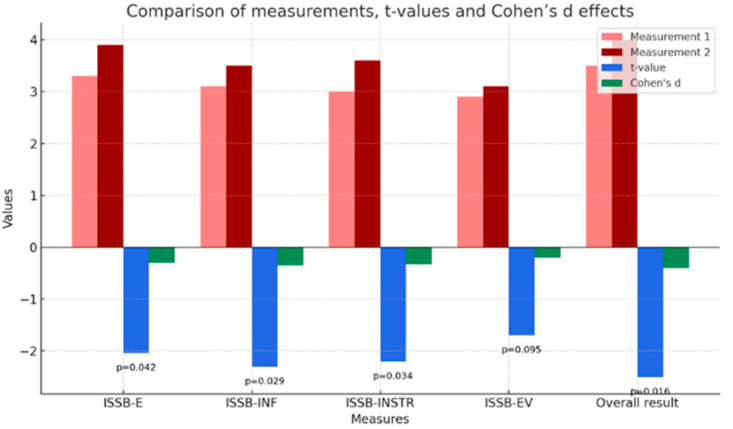
Comparison of measurements, t-values and Cohen’s d effects for the results of the ISSB test (social support) in group A1—group sessions once a week.

Figure 1 presents a more detailed comparison of the measurement results, *t*-test values, and Cohen’s d effect for the above five variables. In each category, the post-intervention measurement values are statistically higher than the pre-intervention measurement values, indicating an increase in the receipt of social support after participation in the study. These results suggest a beneficial effect of interactions with other experimental participants on the amount of social support received by the subjects. Cohen’s d value is negative, indicating a moderate effect size. The largest difference was in the overall score.

Table 7 presents the results of the paired-samples *t*-test comparing the various measures of anxiety and avoidance between the two conditions (before and after participation in the study) for the groups participating in the once-weekly group sessions (A1). After participating in the study, participants showed less avoidance of social interactions than before participating in the study. There was a very strong effect on the general avoidance scale, meaning that participants were less likely to avoid social situations than before participating in the study. Social interaction anxiety also decreased significantly after participation in the study. Similarly, anxiety was related to performing activities, but with a smaller effect. The *p* < 0.05 result indicates statistically significant differences.

**Figure 2:**
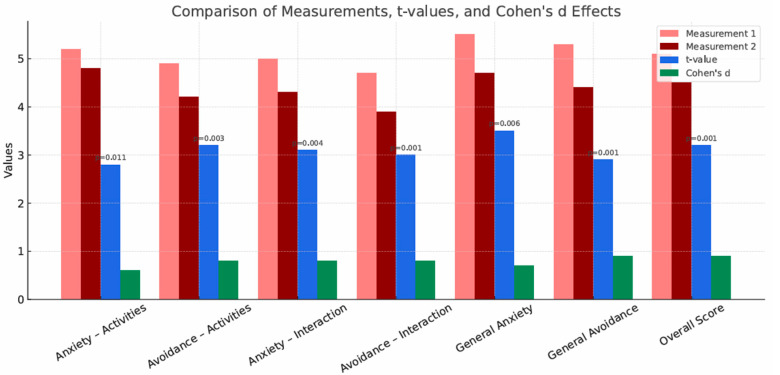
Comparison of measurements, t-values and Cohen’s d effects for the results of the LSAS test (social anxiety) in group A1—group sessions once a week.

Figure 2 presents a comparison of the measurement scores, t values, and Cohen’s d effects for the Liebowitz Social Anxiety Scale measures across participants attending weekly group sessions. In each category, the values in Measurement 2 are lower than in Measurement 1, indicating a decrease in anxiety and avoidance after participation in the study relative to the measurements before participation in the study. This suggests that regular social interaction and the social skills training elements included in such activities had an impact on reducing anxiety related to social situations and reducing the frequency of avoiding them. Cohen’s d values indicate a medium-to-large effect size, which allows us to conclude that there was a significant reduction in anxiety and avoidance. All *p* values are <0.05, which indicates statistically significant differences.

Table 8 presents the results of the dependent samples *t*-test for the measures of the four aspects of social support (emotional support, informational support, instrumental support, and evaluative support) and the total score measure in the two conditions (before and after participation in the study) for individuals participating in the biweekly group sessions. The results do not indicate statistically significant differences between the measures of emotional and instrumental support, but indicate significance at the level of a statistical tendency for the measures of informational and evaluative support. The only significant difference was in the overall score, but the effect size (d = −0.459) suggests only a moderate influence.

**Figure 3:**
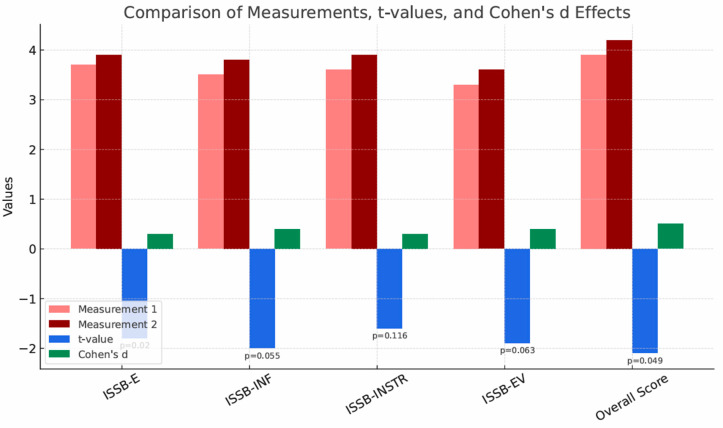
Comparison of measurements, t-values and Cohen’s d effects for the results of the ISSB test (social support) in group A2—group sessions once every two weeks.

Figure 3 presents a comparison of measurement results, t-values, and Cohen’s d effects for the measures of social support. Measurement 2 values are slightly higher than Measurement 1 values, suggesting a small increase in values in the new condition. Cohen’s d effects are small, suggesting a weak effect of the change in condition. Only the overall score shows a significant difference (*p* = 0.049), but the effect is weak. The results for informational and evaluative support indicate significance at the level of statistical tendency (*p* = 0.055, *p* = 0.063). The analysis of these measurements suggests that reducing the frequency of meetings in the group system causes a decrease in the amount of social support received.

Table 9 presents the results of the Wilcoxon signed-rank test for dependent samples, which compares the various measures of anxiety and avoidance between the two conditions (pre- and post-study) for participants attending the biweekly group sessions. This test is an alternative to the *t*-test when the assumption of normality is not met. Each variable showed significant differences between conditions. The largest difference was for the measure ‘Avoidance—Activities’ (r = 1.000). All participants avoided social situations more on Measurement 1 than on Measurement 2. All *p* values are less than 0.001, indicating strong differences. Rank correlations were high (0.900–1.000), indicating strong effects of changing the conditions. The post-intervention measurement condition was associated with significant decreases in anxiety and avoidance across both activities and interactions.

**Figure 4:**
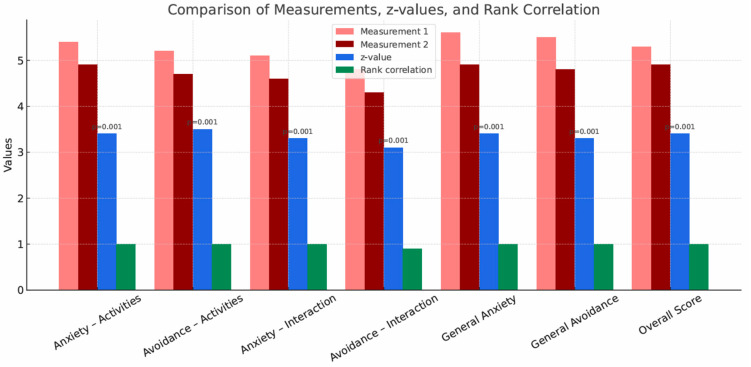
Comparison of measurements, t-values and Cohen’s d effects for the results of the LSAS test (social anxiety) in group A2—group sessions once every two weeks.

Figure 4 presents a comparison of the measurement scores, t values, and Cohen’s d effects for the Liebowitz Social Anxiety Scale measures across participants attending biweekly group sessions. In each category, Measurement 2 values are lower than Measurement 1, indicating a reduction in anxiety and avoidance in the post-intervention condition. These results suggest that participation in RPGs in a group setting is still associated with reduced anxiety and avoidance in participants despite the reduced frequency of encounters. The z-test values are high, indicating statistically significant differences, and the rank correlations are relatively high, suggesting consistency of the results. All *p* values are <0.001, indicating highly significant differences between conditions.

## 4. Discussion

This paper analyzes the impact of participation in RPG sessions on the level of social anxiety and received social support. The study aimed to determine whether regular participation in such activities can contribute to reducing the level of social anxiety and increasing the amount of social support received in people with generalized social phobia. The study was conducted according to a methodological procedure involving two experimental groups. The groups participated in RPG sessions in different conditions: A1 (group meetings once a week) and A2 (group meetings once every two weeks).

The level of social anxiety in those participating in RPG sessions decreased, with a greater decrease observed in those participating in the weekly group system (24%). Cohen’s d effect values in this group indicate a large effect size (d > 0.8). In terms of social support, a higher increase was seen in those participating in the biweekly group (29%), suggesting that regular social interactions in a group may enhance feelings of support.

*p* values < 0.05 for all key comparisons indicate statistical significance of the results, confirming the effectiveness of the RPG intervention in reducing social anxiety and increasing support. The analysis of the collected data shows that participants reacted differently to participating in RPG sessions. Individuals with higher levels of social anxiety at baseline reported some difficulty in fully engaging in narrative interactions, but their comfort gradually increased.

The results of the analyses conducted clearly indicate a significant improvement in the level of social anxiety experienced in all analyzed conditions. The statistical significance of the results in all cases indicates a consistent effect and suggests that the mechanisms present in RPG sessions—such as role-playing, safe exploration of social interactions, and gradual building of relationships—may effectively contribute to reducing this type of anxiety. A significant reduction in the level of avoidance of social situations was also found. Positive results indicate the beneficial effect of role-playing games on overcoming the tendency to avoid social contacts.

In the case of received emotional support, significant improvement was noted only in the condition of once-a-week group sessions. This distribution of results allows us to conclude that participation in RPG sessions may improve emotional social support, but this effect is not clear and may depend on additional factors, such as the length of participation, the intensity of the session or the nature of the group.

In the case of informational social support received, improvement was also noted only in the once-weekly group session condition. This suggests that participants of role-playing games gain access to knowledge, information and advice through contact with the group, especially in the first stages of integration, but the effects are greater with a greater frequency of meetings.

Similarly, for instrumental social support, only the once-weekly group session condition showed statistically significant improvement. These results suggest that the impact of participating in RPG sessions on this form of support may be limited or short-lived. It is possible that building a network of instrumental support (e.g., material or practical assistance) requires a longer period of participation and stronger ties than those formed at the initial stage. Analysis of overall social support indicates improvement in both conditions.

The mechanisms underlying these changes can be linked to the nature of the RPG sessions themselves. Collaborative narrative creation fosters a microculture that fosters trust and cooperation [18], and players have the opportunity to experiment with decisions and behaviors they fear in everyday life. This type of “safe exposure” is important for emotional and social learning processes [23]. RPGs can serve as an intermediate step in exposure therapy [20]—players confront their own fears in a controlled environment, which promotes a gradual increase in their sense of safety and agency. Previous studies [15,17] have demonstrated the development of empathy, improved problem-solving skills, and increased self-esteem as a result of participating in role-playing games. The reduction in social avoidance observed in this study can therefore be interpreted as a result of repeated practice of interpersonal behaviors in a narrative context—consistent with the concept of social skills training [19]. Participants gradually increased their engagement, which can be linked to the development of trust and openness [18]. It is also worth noting that the phenomenon of the “interpenetration” of the game world and reality, described earlier by Polkinghorne [18], could additionally contribute to the consolidation of positive changes—participants often related the behavior of their characters to their own experiences, which could serve as a metaphorical decision-making training.

The results of this study are consistent with previous literature reports. The research conducted by Kipper and Ritchie [24] indicates the therapeutic potential of RPGs, especially in terms of strengthening self-esteem and reducing anxiety in social situations. Similarly, Ott [11] pointed out the usefulness of this method in developing skills to cope with anxiety. Henrich and Worthington’s [25] research suggests that participation in RPGs may have a beneficial effect on cognitive processes and emotion regulation. Additionally, Olivares-Olivares et al.’s [7] research has shown that social skills training combined with group interaction significantly reduces symptoms of social anxiety. Lees [26], in his work, indicated a clear improvement in socialization skills associated with participation in RPG-type role-playing games and the possibility of using this element as a basis for combating social anxiety. In addition, Abbott et al. [17] indicate an increase in the self-confidence and confrontation skills of people participating in RPG-type role-playing games. It is worth noting, however, that compared to previous studies, this experiment included people who had no previous contact with RPGs, which makes the obtained results particularly interesting in the context of first contact with this type of activity.

Despite the general consistency of the results with the literature, some discrepancies were observed. In some cases, no significant changes in the level of social anxiety were observed, which may be due to individual differences between participants, including the level of motivation, personality, or the intensity of the experienced anxiety. It is also possible that the duration of the study was too short to observe stability in the maintenance of the changes that occurred. For example, the research of Poniah and Hollon [27] suggests that exposure alone is not enough, and long-term reinforcement of the effects of therapy through additional behavioral techniques is crucial.

The study had several significant limitations that should be considered when interpreting the results. Primarily, the study sample size was relatively small, limiting the generalizability of the results to a broader population. Furthermore, the lack of a control group makes it difficult to clearly determine whether the observed changes were a true effect of the intervention or could have been due to other factors. Another limitation was the lack of a repeat measurement at least three months after the intervention, which prevented assessment of the sustainability of the achieved effects over time. The low randomness in participant selection and the need to divide participants into subgroups based on availability and willingness to participate may also have impacted the study’s internal reliability. The difficulty in recruiting volunteers stemmed from the fact that participation required a three-month commitment, further limiting the representativeness of the sample.

Taking into account the obtained results, it is worth considering several directions for future research. First of all, the duration of the intervention should be extended to better assess the long-term effects of participation in RPGs. Moreover, an important extension could be to include a comparison of the effects of RPGs with other forms of social skills training, such as classic psychoeducational workshops or cognitive–behavioral therapy. It is also worth considering examining the influence of moderating factors, such as the level of neuroticism or the specificity of the social experiences of the respondents. It is also worth considering re-testing participants several months after completing the study to monitor the maintenance of the effects of participation.

In summary, the results of this study indicate the potential effectiveness of RPGs as an intervention to reduce social anxiety and enhance social support. The results provide a basis for further research in this area and suggest the possibility of implementing RPGs as a complementary therapeutic tool for people with anxiety disorders.

Although the study was not a fully randomized controlled trial, the reporting structure followed the CONSORT 2025 [28] recommendations to ensure transparency and completeness. The CONSORT checklist is available in the Supplementary Materials.

## 5. Conclusions

The study confirmed that participation in RPG sessions can preliminarily effectively reduce social anxiety and enhance the sense of social support in people with non-generalized social phobia. The greatest decrease in anxiety was observed with weekly meetings, while an increase in social support was evident in both conditions, although to varying degrees. The effects were statistically significant, which confirms the preliminary effectiveness of the intervention. Participants gradually increased their engagement in the interactions, indicating the potential of RPGs as a tool to support the breaking down of social barriers. Role-playing games can therefore act as a bridge between therapy and real life, offering a space for experimenting with behavior, making decisions, and building relationships in an atmosphere of acceptance and safety. The results are consistent with previous studies and suggest that RPGs may be a valuable complement to traditional forms of therapy.

## Figures and Tables

**Table 1:** Frequency table for gender, age and course.

Frequency for Gender				
Gender	Frequency	Percent	Valid Percentage	Cumulative Percentage
Women	13	43.33	43.33	43.33
Men	17	56.67	56.67	100.00
**Frequency for Age**				
**Age**	**Frequency**	**Percent**	**Valid Percentage**	**Cumulative Percentage**
18	2	6.67	6.67	6.67
20	3	10.00	10.00	16.67
21	5	16.67	16.67	33.33
22	2	6.67	6.67	40.00
23	9	30.00	30.00	70.00
24	5	16.67	16.67	86.67
25	3	10.00	10.00	96.67
28	1	3.33	3.33	100.00
**Frequency for Course**				
**Course**	**Frequency**	**Percent**	**Valid Percentage**	**Cumulative Percentage**
1	6	20.00	20.00	20.00
2	5	16.67	16.67	36.67
3	1	3.33	3.33	40.00
4	1	3.33	3.33	43.33
5	3	10.00	10.00	53.33
6	5	16.67	16.67	70.00
7	2	6.67	6.67	76.67
8	1	3.33	3.33	80.00
9	1	3.33	3.33	83.33
10	1	3.33	3.33	86.67
11	1	3.33	3.33	90.00
12	3	10.00	10.00	100.00

**Table 2:** Descriptive statistics for the ISSB test (social support) before the intervention.

Group	Valid	Missing	M	Mdn	SD	Skewness	W (Shapiro–Wilk)	*p*
A1	15	0	27.12	27.00	6.55	−0.30	0.98	0.79
A2	15	0	32.50	32.00	8.02	0.10	0.97	0.45

**Table 3:** Descriptive statistics for the ISSB test (social support) after the intervention.

Group	Valid	Missing	M	Mdn	SD	Skewness	W (Shapiro–Wilk)	*p*
A1	15	0	28.90	29.00	6.95	0.05	0.96	0.12
A2	15	0	34.90	34.00	8.10	0.02	0.98	0.74

**Table 4:** Descriptive statistics for the Liebowitz scale (social anxiety) before the intervention.

Group	Valid	Missing	M	Mdn	SD	Skewness	W (Shapiro–Wilk)	*p*
A1	15	0	14.80	15.00	3.30	−0.18	0.97	0.51
A2	15	0	12.10	12.00	3.95	0.72	0.93	0.02

**Table 5:** Descriptive statistics for the Liebowitz scale (social anxiety) after the intervention.

Group	Valid	Missing	M	Mdn	SD	Skewness	W (Shapiro–Wilk)	*p*
A1	15	0	11.40	10.50	4.85	−0.16	0.96	0.15
A2	15	0	8.50	8.00	4.65	0.68	0.94	0.02

**Table 6:** *t*-test for dependent samples—ISSB (social support)—in group A1—group sessions once a week.

Pre-Intervention Measurement	Post-Intervention Measurement	t	df	*p*	Cohen’s d	Cohen’s d SE
emotional support	emotional support	−1.858	14	0.042	−0.480	0.177
informational support	informational support	−2.068	14	0.029	−0.534	0.318
instrumental support	instrumental support	−1.971	14	0.034	−0.509	0.320
evaluative support	evaluative support	−1.382	13	0.095	−0.369	0.283
overall result	overall result	−2.395	14	0.016	−0.618	0.259

**Table 7:** *t*-test for dependent samples—LSAS (social anxiety)—in group A1—group sessions once a week.

Pre-Intervention Measurement	Post-Intervention Measurement	t	df	*p*	Cohen’s d	Cohen’s d SE
fear of activity	fear of activity	2.576	14	0.011	0.665	0.387
avoiding activities	avoiding activities	3.219	14	0.003	0.831	0.389
fear of interaction	fear of interaction	3.049	14	0.004	0.787	0.361
avoiding interactions	avoiding interactions	3.615	14	0.001	0.934	0.418
general anxiety	general anxiety	2.925	14	0.006	0.755	0.410
general avoidance	general avoidance	3.677	14	0.001	0.949	0.498
overall result	overall result	3.673	14	0.001	0.948	0.482

**Table 8:** *t*-test for dependent samples—ISSB (social support), group A2—group sessions once every two weeks.

Pre-Intervention Measurement	Post-Intervention Measurement	t	df	*p*	Cohen’s d	Cohen’s d SE
emotional support	emotional support	−1.335	14	0.102	−0.345	0.240
informational support	informational support	−1.709	14	0.055	−0.441	0.200
instrumental support	instrumental support	−1.248	14	0.116	−0.322	0.217
evaluative support	evaluative support	−1.623	14	0.063	−0.419	0.230
overall result	overall result	−1.778	14	0.049	−0.459	0.202

**Table 9:** Wilcoxon signed-rank test—LSAS (social anxiety)—in group A2—group sessions once every two weeks.

Pre-Intervention Measurement	Post-Intervention Measurement	W	z	*p*	Two-Tailed Rank Correlation	Two-Tailed Rank Correlation SE
fear of activity	fear of activity	119.00	3.351	<0.001	0.983	0.285
avoiding activities	avoiding activities	120.00	3.408	<0.001	1.000	0.285
fear of interaction	fear of interaction	103.50	3.202	<0.001	0.971	0.294
avoiding interactions	avoiding interactions	114.00	3.067	0.001	0.900	0.285
general anxiety	general anxiety	119.00	3.351	<0.001	0.983	0.285
general avoidance	general avoidance	118.00	3.294	<0.001	0.967	0.285
overall result	overall result	119.00	3.351	<0.001	0.983	0.285

## Data Availability

The data presented in this study are available on request from the corresponding author. The data are not publicly available due to participant privacy.

## Supplementary Materials

The following supporting information can be downloaded at: https://www.mdpi.com/article/10.3390/brainsci15111158/s1, File S1: CONSORT_2025_editable_checklist. File S2: Post Hoc.

## Abbreviations

The following abbreviations are used in this manuscript:

RPG	Role-Playing Game
TTRPG	Tabletop Role-Playing Game
SST	Social skills training
LSAS	Liebowitz Social Anxiety Scale
ISSB	Inventory of Socially Supportive Behaviors
