# Supplementary material for: Impact of Participation in Role-Playing Game (RPG) Sessions on the Perceived Level of Social Anxiety and Received Social Support
Source: Brain Sci. 2025 Oct 28;15(11):1158. doi: 10.3390/brainsci15111158 (PMC12650447; doi:10.3390/brainsci15111158)
Supplement: Supplementary file 1 [file brainsci-15-01158-s001.zip › Post Hoc.pdf]

Post Hoc Power Analysis – RPG, Social Anxiety, and Social Support

The analysis was conducted based on data from Tables 8–11 of the article *"Impact of Participation in Role-Playing Game (RPG) Sessions on the Perceived Level of Social Anxiety and Received Social Support"*. Dependent-sample t-tests (df = 14, n = 15) and Wilcoxon tests for group A2 were considered.

Analysis assumptions

- Significance level α = 0.05 (two-tailed test)
- Sample size: n = 15 per group
- Effect size indicators: Cohen's d (for t-tests) and r coefficient (for Wilcoxon test)
- Post hoc power calculated based on d and n

Group A1 – ISSB (social support)

Indicator	Cohen's d	Power (1–β)
Emotional support	0.48	0.43
Informational support	0.53	0.47
Instrumental support	0.51	0.45
Evaluative support	0.37	0.30
Overall result	0.62	0.58

Power values range from 0.30 to 0.58, indicating moderate test strength. The probability of a Type II error (β) ranges from 0.4 to 0.7.

Group A1 – LSAS (social anxiety)

Indicator	Cohen's d	Power (1–β)
Anxiety - activity	0.67	0.63
Avoidance - activity	0.83	0.79
Anxiety - interaction	0.79	0.76
Avoidance - interaction	0.93	0.88
General anxiety	0.76	0.74
General avoidance	0.95	0.90

Overall result	0.95	0.90

Power values ranging from 0.63 to 0.90 indicate good to high test strength. Effects in this group are statistically robust.

Group A2 – ISSB (social support)

Indicator	Cohen's d	Power (1–β)
Emotional support	0.35	0.23
Informational support	0.44	0.36
Instrumental support	0.32	0.20
Evaluative support	0.42	0.34
Overall result	0.46	0.38

Power is low (0.20–0.38), suggesting that nonsignificant results may be due to a small number of participants. To detect effects of approximately $d \approx 0.45$, a sample of $n \approx 35–40$ would be needed.

Group A2 – LSAS (social anxiety, Wilcoxon test)

For all indicators, $r \geq 0.9$, corresponding to $d \approx 2.6–3.0$. Estimated power is ≈ 1.00 (100%), indicating a very strong effect.

Summary

- Group A1 (ISSB): moderate power (0.30–0.58)
- Group A1 (LSAS): good–high power (0.63–0.90)
- Group A2 (ISSB): low power (0.20–0.38)
- Group A2 (LSAS): very high power (~1.00

Results suggest that tests related to social anxiety had sufficient power, whereas measurements of social support—particularly in the group with less frequent sessions—would require a larger number of participants.
